# Cytolytic toxin production by *Staphylococcus aureus* is dependent upon the activity of the protoheme IX farnesyltransferase

**DOI:** 10.1038/s41598-017-14110-8

**Published:** 2017-10-23

**Authors:** Emily Stevens, Maisem Laabei, Stewart Gardner, Greg A. Somerville, Ruth C. Massey

**Affiliations:** 1 Milner Centre for Evolution, Dept. of Biology and Biochemistry, University of Bath, Bath, UK; 2 Microbiology Department, Hospital Universitari Germans Trias i Pujol, Institut d’Investigació Germans Trias i Pujol, Universitat Autònoma de Barcelona, Badalona, Spain; 3 School of Veterinary Medicine and Biomedical Sciences, University of Nebraska-Lincoln, Nebraska, USA; 4 School of Molecular and Cellular Medicine, University of Bristol, Bristol, UK

## Abstract

*Staphylococcus aureus* is a medically important pathogen with an abundance of virulence factors that are necessary for survival within a host, including the production of cytolytic toxins. The regulation of toxin production is mediated by the Agr quorum sensing system, and a poorly defined post-exponential growth phase signal independent of Agr. As part of a recent genome wide association study (GWAS) to identify novel loci that alter the expression of cytolytic toxins, a polymorphism in the *cyoE* gene, which encodes a protoheme IX farnesyltransferase, was identified. This enzyme is essential for processing heme into the electron transport chain for use as an electron acceptor. Interestingly, without this enzyme *S. aureus* were repressed in their ability to secrete cytolytic toxins, and this appears to be mediated through repression of the Agr quorum sensing system. We hypothesize that the loss of electron transport is inducing feedback inhibition of metabolic capabilities that suppress the TCA cycle, and that this coupled with decreased RNAIII transcription prevents synthesis of cytolytic toxins.

## Introduction

*Staphylococcus aureus* is a commensal bacterium that persistently colonises the nasal passages of approximately 20% of the human population^1,2^. As an opportunistic pathogen, it commonly infects people whose immune system is compromised through illness, injury or age. These infections vary widely in terms of anatomic site and disease severity, ranging from minor skin and soft tissue infections to life threatening pneumonia or bacteraemia^1,2^. Central to *S. aureus*’ ability to survive the host immune response is the synthesis of numerous virulence determinants that help facilitate nutrient acquisition and immune evasion. Specifically, *S. aureus* produces adhesins that allow it to adhere to and colonise host tissues; proteins and a capsule that facilitate evasion of the host immune system; and secreted toxins that damage host cells and release nutrients. The expression of these virulence determinants is in part regulated by the Agr quorum sensing system, with a poorly defined post-exponential growth phase signal independent of Agr^3^. The tricarboxylic acid (TCA) cycle activity^4^ is also critical for cytolytic activity where TCA cycle mutants have decreased synthesis of secreted toxins. In staphylococci, TCA cycle activity is induced during the post-exponential growth phase, providing the bacterium with carbon, energy, and reduced dinucleotides^5^. TCA cycle-derived four- and five-carbon intermediates are used in biosynthetic reactions to synthesize precursors (e.g. amino acids); while energy (e.g. ATP) drives many critical cellular processes and reduced dinucleotides can donate electrons to the electron transport chain to generate ATP by oxidative phosphorylation.

Recently we analysed genetic polymorphisms within *S. aureus* clinical isolates from the major hospital-associated MRSA lineage ST239^6^, and found there was little variability in their adhesive capabilities, but there was significant variation in cytolytic toxin production^7^. The application of GWAS (genome wide association studies) to this data identified a number of polymorphic loci that were statistically associated with altering cytolytic toxin synthesis. One of those loci, *cyoE*, encodes the enzyme protoheme IX farnesyltransferase, an enzyme involved in catalysing the conversion of heme B to heme O^8,9^. Heme O is incorporated into the electron transport chain as an electron acceptor, facilitating aerobic respiration and energy production^10,11^. As mentioned, TCA cycle mutants have decreased accumulation of cytolytic toxins; hence, the connection between the electron transport chain and the TCA cycle suggests these variants may have decreased TCA cycle activity and toxin accumulation. To address these possibilities, the metabolism and phenotype of a *cyoE*-deficient mutant and complemented strains were analysed.

## Materials and Methods

### Strains and cultivation conditions for toxicity assays

The Δ*cyoE* mutant (NE1434) was obtained from the Nebraska Transposon Mutant Library, which is a collection of 1,952 *S. aureus* mutants in the USA300 strain JE2^12^. The USA300 JE2 wild type was used as a control in all assays. Bacterial strains were grown at 37 °C in brain heart infusion broth (BHI; Oxoid) or on tryptic soy agar (TSA; Sigma). When needed, erythromycin (5 µg/ml), chloramphenicol (10 µg/ml) and tetracycline (50 ng/ml) were added.

### Growth, pH and acetic acid assays

Unless stated otherwise, bacterial strains were grown in filter-sterilized tryptic soy broth (TSB; Becton Dickinson and Company) and cultivated at 37 °C, with 225 rpm aeration, and using a flask-to-medium ratio of 10:1. Bacterial pre-cultures were prepared from overnight cultures diluted 1:100 in TSB and incubated for 1.5 to 2 h. These pre-cultures were centrifuged for 5 min at 5,000 rpm, and the exponentially growing cells were inoculated into pre-warmed TSB to an optical density at 600 nm (OD_600_) of 0.02. No antibiotics were used in this assay. Cultures were diluted prior to reading the density at the later stages of growth to avoid any saturation effects in the spectrophotometer. The pH of the culture medium was determined hourly using an Accumet AR60 pH meter (Fisher Scientific). The acetic acid assays were performed on culture supernatants (1 ml) that were harvested hourly by centrifugation and the acetate and glucose concentrations were determined with kits purchased from R-Biopharm and used according to the manufacturer’s protocol.

### Toxicity assay

THP-1 cells are an immortal monocytic cell line^13^ that is sensitive to 13 of the 15 toxins produced by *S. aureus* that are present in the bacterial supernatant^7^. They are continuously sub-cultured at 2–3 day intervals in a solution of RPMI 1640 containing fetal bovine serum and an antibiotic solution of 200mM L-glutamine, 10,000 units of penicillin and 10 mg/ml streptomycin. Following overnight growth in BHI broth, the bacterial cultures were centrifuged for 10 minutes at 10,000–12,000 × g and the supernatant was harvested. The supernatant was diluted to a 30% vol/vol in BHI broth and 20 µl of this was added to 20 µl of washed THP-1 cells at a concentration of 120–150 cells per 1 µl, and incubated for 12 minutes at 37 °C. Following incubation of bacterial supernatant with THP-1 cells, samples were stained with 260 µl Guava ViaCount reagent, incubated at room temperature for 5 minutes, and loaded onto a Guava flow cytometer to determine the percentage of THP-1 cell death in each sample.

### Complementation of Tn mutant

To confirm that loss of the *cyoE* gene was responsible for the observed loss in toxicity, the wild-type *cyoE* gene was re-introduce into the transposon mutant. The plasmid vector pRMC2 was used because it contains a tetracycline-inducible promoter region that allows transcription of the gene of interest to be controlled. The wild-type *cyoE* gene was amplified by PCR using the following primer sequences:

*cyoE* FW: GCTGGTACCATGAACAAATTTAAGGAG; *cyoE* RV: GCGAATTCAATTTCATCCTAACTTAATT

Restriction enzyme sites for KpnI and EcoR1 were added to the forward and reverse primers, respectively. The *cyoE* gene and plasmid pRMC2 were then digested with KpnI and EcoR1 and the resultant products were ligated using T4 DNA Ligase. Successfully ligated plasmids containing the wild-type *cyoE* gene were transformed into *E. coli* DH5α competent cells through electroporation, plasmid DNA was isolated and passaged through *S. aureus* RN4220, before finally being transformed into the strain JE2 Δ*cyoE* transposon mutant NE1434. For transformation through electroporation, bacteria were cultivated in BHI liquid culture to an OD_(550)_ of 0.2–0.3 and washed four times in ice cold 0.5 M sucrose. After the final wash bacteria were suspended in 100 µl of 0.5 M sucrose before being added to 1–5 µg/ml of DNA. Bacteria were incubated on ice with the DNA for 20 minutes and electroporated in 0.2 cm cuvettes for 4.2–4.6 milliseconds. Following electroporation, 800 µl BHI was added to the cuvettes and incubated for 1 hour at 37 °C without shaking. The transformants were then plated on TSA containing 10 µg/ml chloramphenicol, and for the transposon mutant strain 5 µg/ml erythromycin was also added to the agar.

### Phage transduction of ΔcyoE from strain JE2 into strain SH1000

Donor cells were inoculated into liquid culture from single colonies and grown overnight, and the following day 200 µl of this culture was added to 25 ml BHI containing 250 µl 1 M MgSO_4_ and 250 µl 1 M CaCl_2_. This was grown for one hour and then 100 µl phage 11 was added to the culture and grown for a further four hours minimum. Supernatant was obtained from this culture through centrifugation (12,000 × g for 3 minutes), and was then filter sterilised. Optimal plaque titre was in the range of 10^7–10^. Next, recipient cells were grown overnight in 20 ml LK broth (1% tryptone, 0.5% yeast extract, 0.7% potassium chloride), then this culture was centrifuged (2,500 × g for 10 minutes) and the pellet suspended in 1 ml LK broth. To 250 µl of recipient cells was added 500 µl LK broth plus 10 mM CaCl_2_ and 250 µl of the phage lysate from the previous step. This culture was incubated statically for 25 minutes and then with shaking at 180 rpm for 15 minutes. 500 µl ice cold 0.02 M sodium citrate was then added, and the culture centrifuged at 10,000 × g for 10 minutes. The pellet was suspended in 500 µl 0.02 M sodium citrate and left on ice for 2 hours. 100 µl of this was then plated neat on LKA plates containing 0.02 M sodium citrate and selective antibiotic, and this was incubated for at least 20 hours at 37 °C.

### RNAIII activity assay

A plasmid containing a GFP-tagged copy of the RNAIII gene was transformed into strains USA300 JE2, Δ*cyoE*, RN6390B (an *agr*-positive strain) and RN6911 (an *agr*-negative strain). Single colonies were then inoculated into BHI as described above for liquid overnight cultures, and the following morning a 1:10,000 dilution was made into BHI at a flask to medium ratio of 10:1. Strains were cultured at 37 °C and aerated at 180 rpm; OD_(600)_ and GFP fluorescence (485/520) readings were then taken at hourly intervals over 12 hours.

### Aconitase activity

Bacteria were harvested during the postex-ponential growth phase (6 h) by centrifugation, suspended in ACN buffer (100 M fluorocitrate, 90 mM Tris/HCl, pH 8.0), and lysed with lysing matrix B tubes and a FastPrep instrument (MP Biomedicals). The lysate was centrifuged for 5 min at 13,200 rpm at 4 °C, and the aconitase activity in the cell-free lysate was measured by the method of Kennedy *et al*.^14^. One unit of aconitase activity is defined as the amount of enzyme necessary to give a A240 min^−1^ of 0.0033.

### Western blot for α-toxin

Proteins were precipitated from bacterial supernatant following 18 hrs of growth using trichloroacetic acid (TCA) at a final concentration of 20% for 1 hour on ice. Samples were then washed three times using ice cold acetone, and solubilised in 100 µl 8 M urea. 20 µl of each sample was mixed with 20 µl loading dye, and heated at 100 °C for 2 minutes. 10 µl of each sample was then subjected to 10% SDS-PAGE and separated proteins were electroblotted onto a nitrocellulose membrane using a semi-dry blotter at 15 V for 30 minutes (BioRad). Membranes were blocked overnight using 3% BSA in PBS-T (containing 0.1% Tween), and were then incubated for 1 hour with rabbit polyclonal antibodies specific for α-toxin. After washing 3 times for 5 minutes with PBS, membranes were incubated for another hour with horseradish peroxidase-coupled Protein G. All incubation steps were done at room temperature. Membranes were washed twice for 20 minutes in PBS, and blots were then visualised using an Opti-4CN detection kit. Band intensities were quantified using ImageJ (v 1.46r).

### PSM quantification

Overnight cultures were diluted 1:1000 in 50 ml BHI and grown for 18 hours at 37 °C with shaking (180 rpm). 30 ml supernatant was added to 10 ml 1-butanol and these samples were incubated for 3 hours at 37 °C with shaking. Samples were then centrifuged for 3 minutes and 1 ml of the upper organic phase was collected. Protein samples were concentrated overnight using a SpeedVac and dried samples were then solubilised in 150 µl 8 M urea. Samples were loaded and run on 10% SDS-PAGE as described above and then stained using SimplyBlue SafeStain as per the protocol. Band intensities were quantified using ImageJ (v 1.46r).

### Micro-aerobic environment

To assess the effect of microaerobic growth conditions on toxicity in the wild-type strain JE2, bacteria were cultivated using a flask-to-medium ratio of 10:8 and toxicity assays were conducted using culture supernatant as described above. All other growth conditions remained unchanged.

### Statistics

All of the data presented here was found to be normally distributed, and as such significance (p values) were determined using the Student’s unpaired 2-tailed T-test.

## Results and Discussion

### Association between toxicity and the polymorphic *cyoE* gene

**Figure 1:**
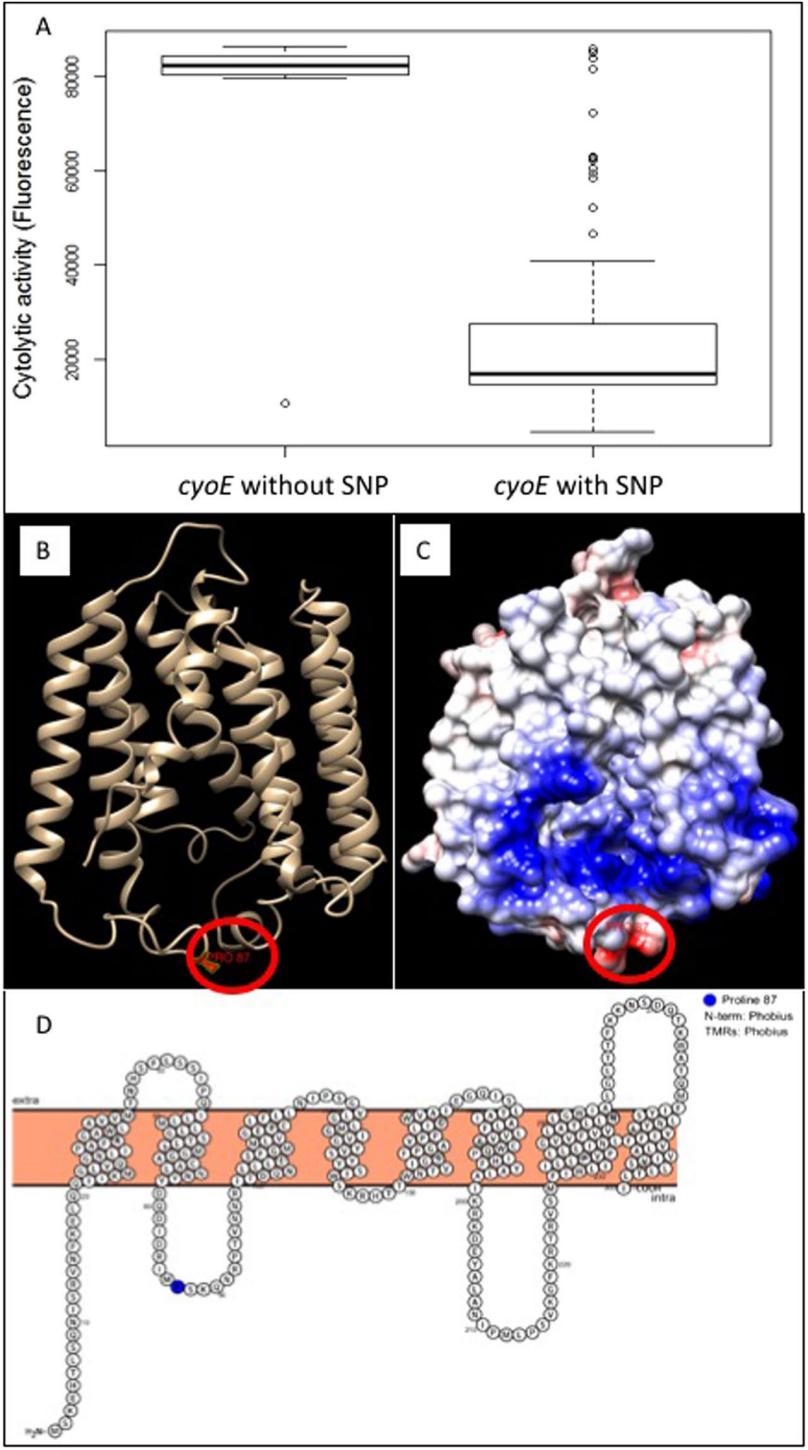
GWAS identified association between mutations in the *cyoE* gene and toxicity. (**A**) Result of a GWAS study on a collection of 90 ST239 MRSA isolates that associated polymorphisms in the *cyoE* gene with the toxicity of the bacteria. The data presented is the mean toxicity (as measured by lysis of fluorescent dye containing vesicles^22^ of the isolates with and without polymorphisms in the gene. Eight isolates contained the TW20 reference *cyoE* gene, and 82 isolates contained the SNP. (**B**) The position of the amino acid change P87L in the protein, protoheme IX farnesyltransferase, encoded by the *cyoE* gene has been mapped to a ribbon model of the structure of this enzyme, and in (**C**) coulorimbic surface colouring has been used to illustrate the white hydrophobic domains of the protein, the blue positively charged amino acid residues, and the red negatively charged amino acid residues. (**D**) A model of the protoheme IX farnesyltransferase situated in the cell membrane, and position of the toxicity associated amino acid change, as modelled by Protter^16^.

A recent GWAS study identified an association between a polymorphic version of the *cyoE* gene with changes in the cytolytic activity (toxicity) of *S. aureus*, suggesting this locus may contribute to toxicity^7^. A comparison of these data has demonstrated that there was a 2.9-fold reduction (Fig. 1A; p = 0.0008) in the mean toxicity of *S. aureus* containing the SNP in the *cyoE* gene relative to those with the *cyoE* gene with no SNPs (i.e. that found in the reference strain of the ST239 lineage, TW20^6^). The *cyoE* gene encodes a protoheme IX farnesyltransferase^8,9^, which is a membrane associated protein involved in the processing of heme, enabling the bacteria to respire aerobically^10,11^.

The SNP change observed in the collection of clinical strains confers a change from proline to leucine at position 87 in the translated product of the *cyoE* gene. To determine the likelihood of this change affecting the activity of this protein we built a model of it using SWISS-MODEL^15^. We then viewed this model in Chimera to visualise where the amino acid change occurred and how it might therefore affect the activity of the protein in the clinical strains (Fig. 1B and C). As this is a membrane protein we also present a model of this which was generated using Protter^16^ (Fig. 1D). Based on these we hypothesise that the change to leucine could give the loop in which it is located more flexibility and make it more hydrophobic, which could result in the loop flipping inwards on itself. The change also appears to be within a region that could be the heme binding site of this enzyme; Fig. 1C shows the suggested structure of this protein with coulorimbic surface colouring to show positively and negatively charged regions of the protein. The blue region in the lower middle of the structure is thought to be the active site of this enzyme, thus any change to the structure of the loop beneath, particularly the loop flipping inwards, could affect the function of the active site. Further analysis of the structure of this protein would be required to confirm this hypothesis.

### Functional verification of the contribution protoheme IX farnesyltransferase makes to *S. aureus* toxicity

**Figure 2:**
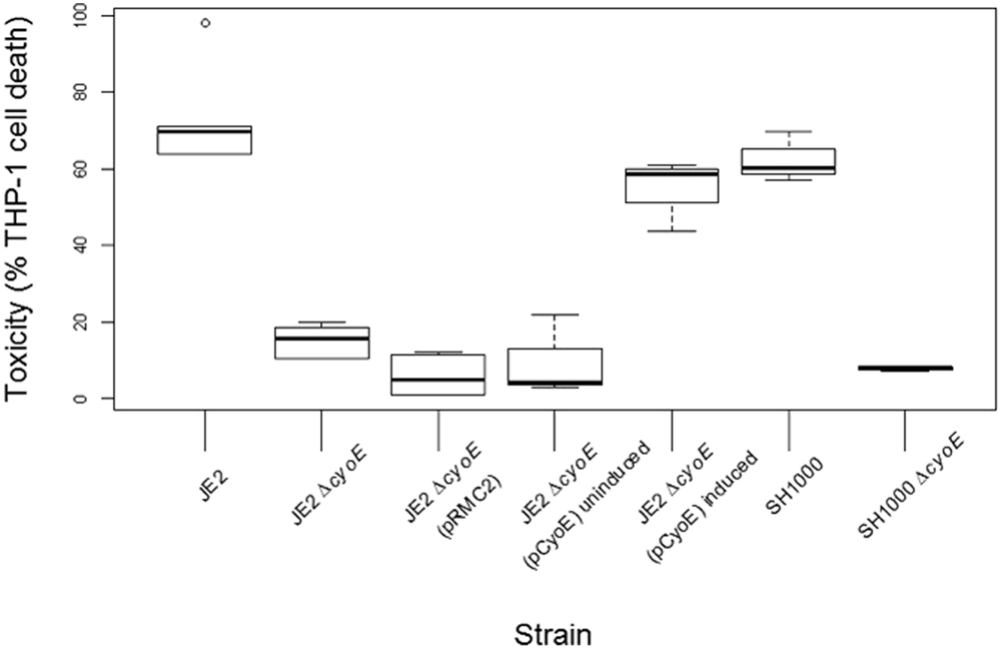
Functional verificaiton of the contribution the *cyoE* gene makes to *S. aureus* toxicity. The *cyoE* gene was inactivated by transposon insertion in both the JE2 and SH1000 backgrounds. In the JE2 background the loss of toxicity was restored by expressing the gene from an inducible promoter on the pRMC2 vector plasmid. Complemenation controls of the empty vector (JE2Δ*cyoE* (pRMC2)), and the vector with the c*yoE* gene cloned, but uniduced (JE2Δc*yoE* (pCyoE)) have been included.

To verify the association between the *cyoE* gene and toxicity, the cytolytic activity of a Δ*cyoE* transposon mutant from the Nebraska Transposon Mutant library and the isogenic strain JE2 were compared. To quantify this, the THP-1 monocytic cell line, which is sensitive to both the Phenol Soluble Modulins (PSMs) and many of the other cytolytic toxins secreted by *S. aureus*, was exposed to culture supernatants and toxicity was assessed. As suggested by the GWAS results, the Δ*cyoE* mutant had significantly decreased toxicity relative to the isogenic wild-type strain (Fig. 2; p < 0.0001). Complementation of the Δ*cyoE* mutant resulted in restoration of toxicity to wild type levels. To ascertain if the effect of inactivating *cyoE* was strain-dependent, the mutation was transduced into *S. aureu*s strain SH1000 and toxicity was assessed. Similar to the strain JE2 background, inactivation of the *cyoE* gene in strain SH1000 caused a significant loss of toxicity (Fig. 2; p = 0.004). Taken together, these data confirm that the *cyoE* gene contributes to the ability of *S. aureus* to produce toxins.

### Protoheme IX farnesyltransferase activity affects the ability to activate the Agr quorum sensing system

**Figure 3:**
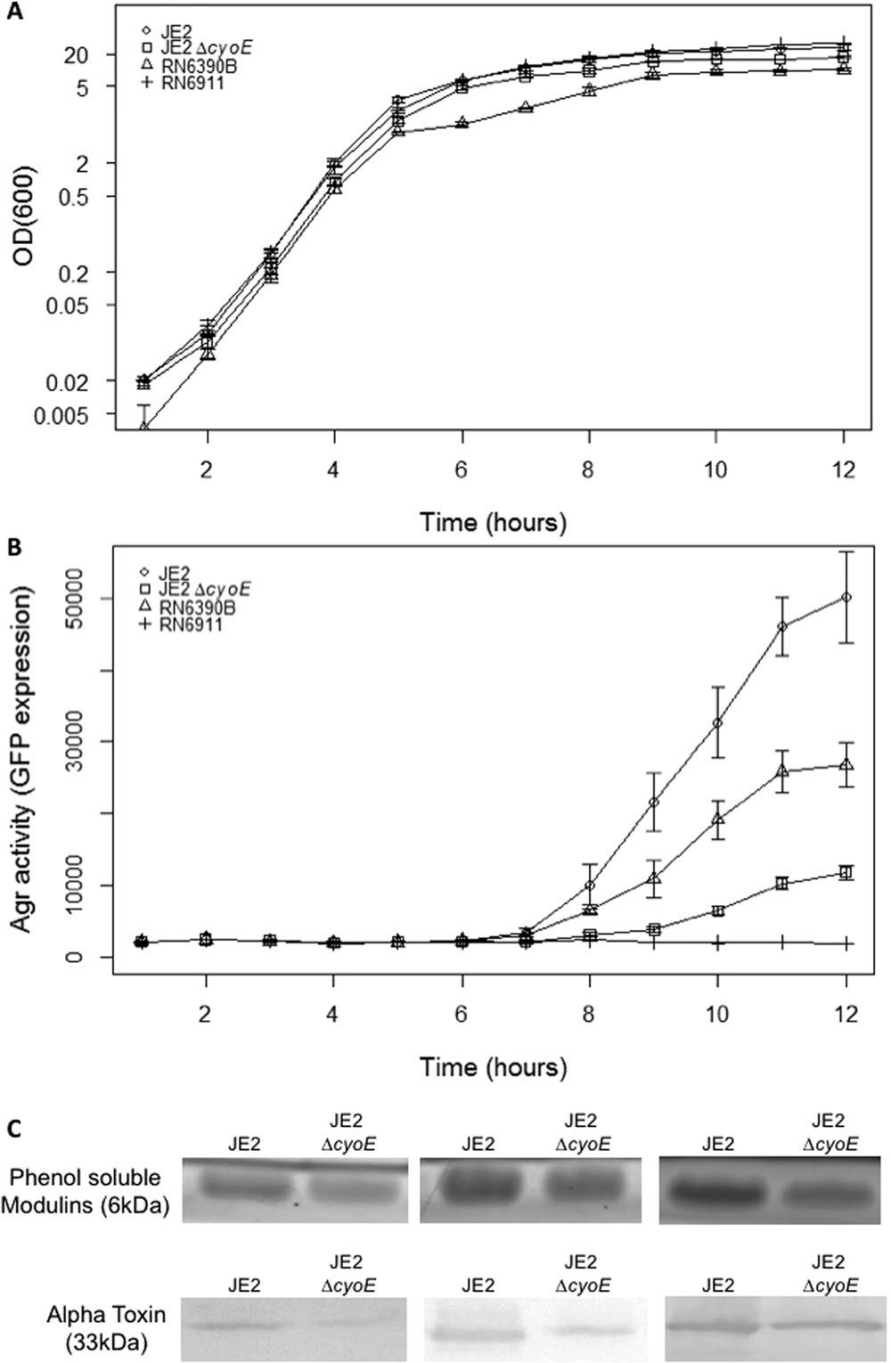
Activation of the Agr quorum sensing system is dependent upon protoheme IX farnesyltransferase activity. (**A**) The growth of strains carrying the RNAIII reporter plasmid was monitored over 12 hours demonstrating that inactivation of the *cyoE* gene has minimal effect on the growth of *S. aureus* during this time frame *in vitro*. (**B**) Expression of Agr was monitored over 12 hours of growth where the inactivation of *cyoE* was found to have a negative effect on Agr activity. Control strains RN6390B (Agr+) and RN6911 (Agr−) have been included for comparison. (**C**) The downstream effect of the loss of Agr activity was verified by comparing the production of alpha toxin and the PSMs by both the wild-type and Δ*cyoE* mutant, where the mutant produced 2.2-fold less alpha toxin and 1.5-fold less PSM.

As the Agr quorum sensing system is a major regulator of toxin synthesis^17–19^ and as the inactivation of *cyoE* dramatically affects toxicity, we hypothesised that this effect of the loss of *cyoE* may be mediated through the Agr system. To test this hypothesis, an RNAIII::*gfp* fusion plasmid, which acts as a reporter of Agr activity, was introduced into the JE2 wild-type and Δ*cyoE* mutant strains and fluorescence was monitored over time (Fig. 3A and B). There was a significant reduction in fluorescence in the Δ*cyoE* mutant (p = 0.025), demonstrating that RNAIII transcription and consequently Agr activation is altered in the Δ*cyoE* mutant relative to the wild-type strain. To further examine the effect of the loss of the *cyoE* gene on Agr activity we quantified the expression of toxins known to be under its regulation, where the secretion of alpha toxin was quantified by western blotting and PSMs by butanol extraction. These assays were performed in triplicate (Fig. 3C) where we found on average there was 2.2-fold more alpha toxin, and 1.5-fold more PSMs expressed by the wild type JE2 strains when compared to the Δ*cyoE* mutant. These results confirm that in the absence of the *cyoE* gene the expression and activity of the Agr quorum sensing system is repressed.

### Protoheme IX farnesyltransferase affects the TCA cycle

**Figure 4:**
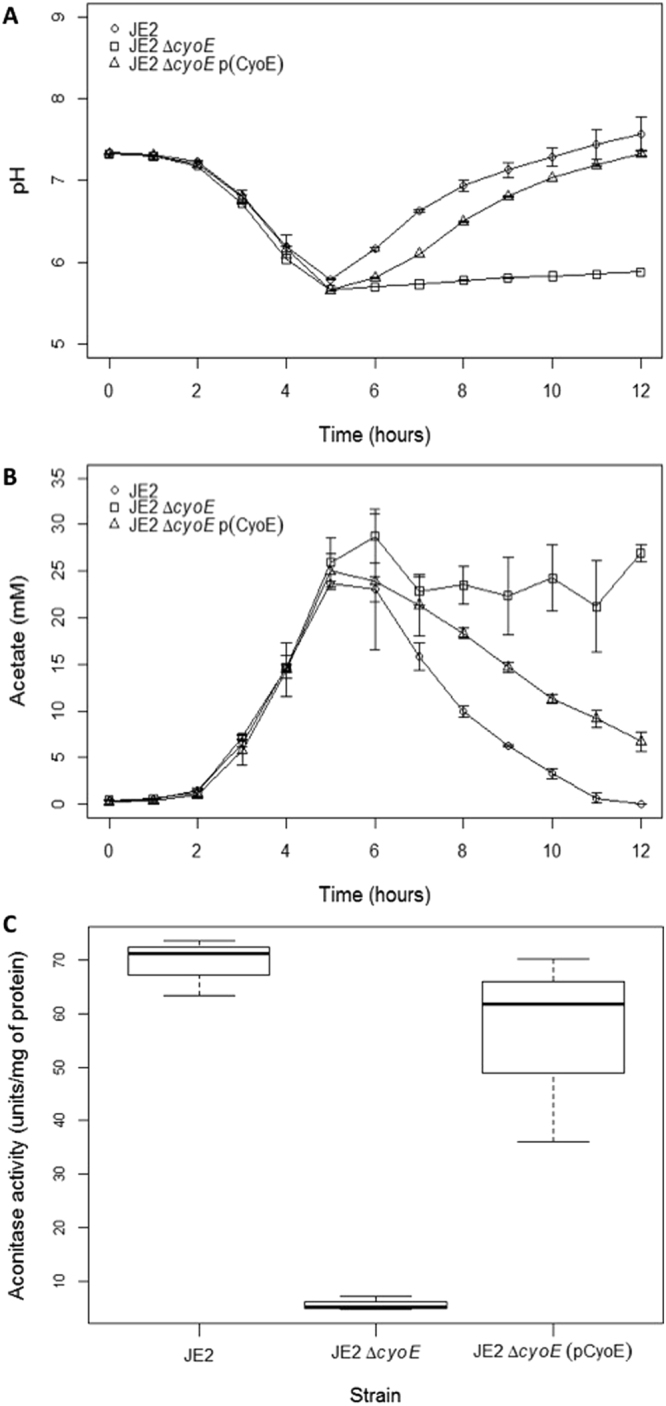
The activity of the TCA cycle is affected by the loss of expression of protoheme IX farnesyltransferase. The pH (**A**) and acetate (**B**) levels of the supernatant of the wild type (JE2), Δ*cyoE* mutant and the complemented mutant were quantified over 12 hours of growth. Inactivation of the *cyoE* gene affected both the pH and acetate levels, the effects of which were complemented by expression of the gene from the plasmid pCyoE. (**C**) Aconitase activity, a key feature of the TCA cycle, was quantified in the wild type (JE2), Δ*cyoE* mutant and complemented mutant. Inactivation of the *cyoE* gene significantly reduced aconitase activity, the effect of which was complemented by expression of the gene from the pCyoE plasmid.

The protoheme IX farnesyltransferase (CyoE) is essential for electron transport in many organisms, and electron transport facilitates the oxidation of reduced dinucleotides that are generated from TCA cycle activity. As such the inactivation of *cyoE* in *S. aureus* should dramatically decrease TCA cycle activity. The decreased Agr activity (Fig. 3) coupled with decreased TCA cycle activity could provide a potential explanation for the decreased cytotoxicity we observed for the Δ*cyoE* mutant, as we have previously shown secretion of cytotoxins requires TCA cycle activity^4^. To test this hypothesis, the catabolism of acetate, which requires TCA cycle activity, was assessed. Acetate accumulates in the culture medium during the exponential growth phase due to the incomplete oxidation of carbohydrates when the TCA cycle is repressed. The catabolism of acetate begins when carbohydrates are depleted and the TCA cycle is de-repressed in the post-exponential growth phase. Loss of a functional TCA cycle will result in a deficiency in acetate catabolism, leading to a build-up of acetate in the culture medium and lowering its pH. As expected, the acidification of the media was equivalent, as was the accumulation of acetate between the wild-type, Δ*cyoE* mutant and complemented strains over the first five hours of growth (Fig. 4A). However, once the bacteria ceased growing exponentially (from 5hr onwards) the Δ*cyoE* mutant failed to alkalinise the culture medium and did not catabolise acetate (Fig. 34B). To specifically verify the effect of *cyoE* on the TCA cycle activity we also quantified aconitase activity, which is a major TCA cycle enzyme catalysing the interconversion of citrate and isocitrate. During the post-exponential growth phase (6hr) we found that the wild-type and complemented mutant strains both had significantly more aconitase activity when compared to the Δ*cyoE* mutant (Fig. 4C; p = 0.001). Taken together, the Δ*cyoE* mutant’s inability to catabolise acetate and alkalinise the culture medium, demonstrates that inactivation of *cyoE* blocks TCA cycle activity.

### Growth of *S. aureus* under micro-aerobic conditions mimics the effect of a loss of *cyoE*

**Figure 5:**
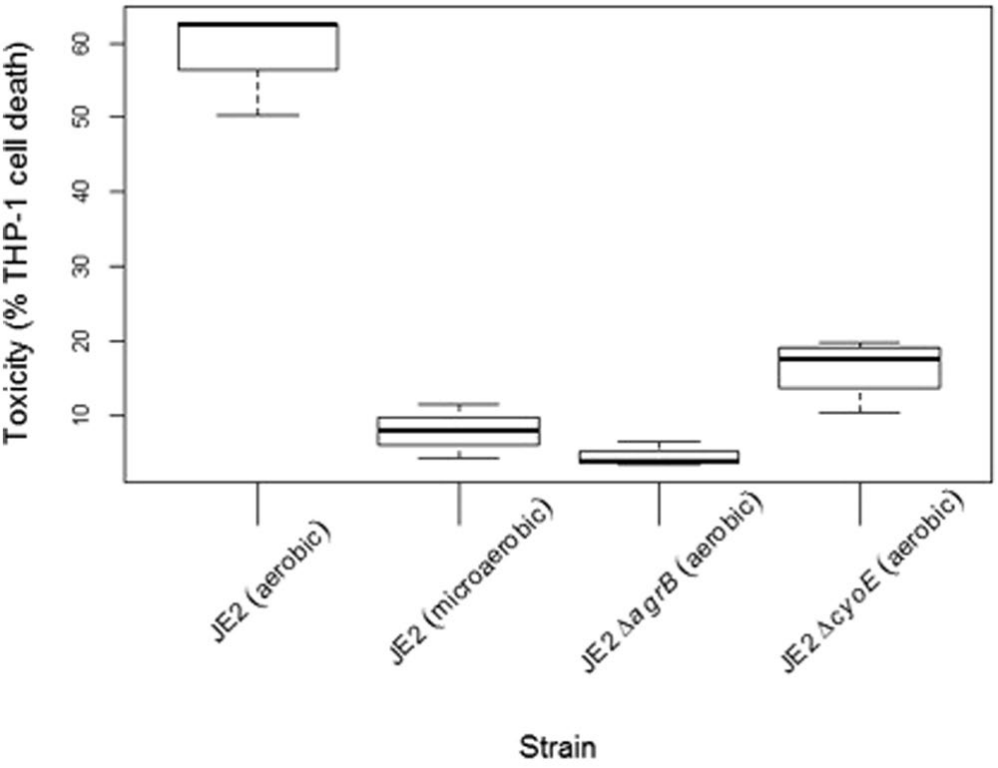
Repression of the TCA cycle by growing *S. aureus* under microaerobic conditions repressed the expression of cytolytic toxins. The wild type (JE2) strain was grown under microaerobic (10:8 flask to broth volume ratio) and under aerobic (10:1 flask to broth volume ratio) to determine the effect this has on TCA cycle activity has on cytolytic toxin activity. An Agr mutant and the Δ*cyoE* mutant both grown aerobically were included. Growth of the wild type strain under the microaerobic conditions had an equivalent effect on toxicity as inactivation of either the Agr system or the *cyoE* gene, demonstrating the contribution the TCA cycle makes to the toxicity of *S. aureus*.

As an alternative means of demonstrating the effect of reducing TCA cycle activity on toxicity, we examined whether an effect equivalent to the inactivation of *cyoE* could be achieved by culturing the bacteria in a microaerobic environment. As a facultative anaerobic bacterium, *S. aureus* can grow under anaerobic conditions, however its growth rate is significantly affected with one study finding a nine-fold difference in final cell density when aerobic and anaerobic growth conditions were compared^20^. This study also found that under anaerobic conditions *S. aureus* secretes less alpha toxin, however these effects on cell growth make assessing the relative levels of expression of quorum-sensing dependent proteins complicated. As such, to repress the TCA cycle while minimising the growth defects associated with anaerobic conditions we created a micro-aerobic environment by growing the bacteria in air, but manipulating the flask-to-medium ratio^21^. We grew JE2 for 18 hrs and the effect of comparing a flask-to-medium ratio of 10:1 and 10:8 on the secretion of toxins was quantified (Fig. 5). The bacteria grown in micro-aerobic conditions had a relatively small (1.9-fold) decrease in biomass when compared to those grown aerobically. There was however a significant effect on toxicity with the bacteria growing in a micro-aerobic environment expressing levels of toxicity equivalent to an aerobically grown Δ*agrB* mutant (Fig. 5).

## Conclusion

*S. aureus* in one of the most sequenced bacterial pathogens, with many thousands of genomes being publicly available. We are at a point in which we can utilise this sequence data to understand the biology of this important pathogen in greater detail. This study began as a GWAS analysis of a collection of ST239 isolates and is completed here as we characterise the molecular detail of the role of a GWAS identified locus on virulence. Here we demonstrate that the *cyoE* gene, which encodes a protoheme IX farnesyltransferase enzyme, plays a critical role in the ability of *S. aureus* to secrete cytolytic toxins. In the Δ*cyoE* mutant, activation of the Agr quorum sensing system is significantly delayed, despite the bacteria reaching sufficient cell density. We believe this effect on the Agr system is coupled to repression of the TCA cycle caused by the loss of heme O production and its role as an electron acceptor in the electron transport chain. This work reiterates the important link between metabolism and virulence in *S. aureus*, but also demonstrates the variability that exists in these attributes amongst clinical isolates causing disease in humans. As genome sequencing becomes more embedded in clinical diagnostic procedures, information relating to such polymorphic loci could be used to assist in the diagnosis of highly virulent infections.
